# Prevalence of sexually transmitted infection in pregnancy and their association with adverse birth outcomes: a case–control study at Queen Elizabeth Central Hospital, Blantyre, Malawi

**DOI:** 10.1136/sextrans-2024-056130

**Published:** 2024-07-23

**Authors:** Charlotte van der Veer, Chifundo Kondoni, Annie Kuyere, Fatima Mtonga, Vita Nyasulu, George Shaba, Chelsea Morroni, Gladys Gadama, Luis Gadama, Kondwani Kawaza, Queen Dube, Neil French, David Lissauer, Bridget Freyne

**Affiliations:** 1 Malawi-Liverpool-Wellcome Trust Clinical Research Programme, Blantyre, Malawi; 2 Department of Children’s and Women’s Health, University of Liverpool, Liverpool, UK; 3 Botswana–Harvard AIDS Institute Partnership, Gaborone, Botswana; 4 University of Edinburgh, Edinburgh, UK; 5 Queen Elizabeth Central Hospital, Blantyre, Malawi; 6 Institute of Infection, Veterinary and Ecological Science, University of Liverpool, Liverpool, UK; 7 Children's Health Ireland, Dublin, Ireland

**Keywords:** NEISSERIA GONORRHOEAE, SYPHILIS, PREGNANCY, Prevalence, Diagnostic Screening Programs

## Abstract

**ABSTRACT:**

**Background:**

There are limited data on the epidemiology of sexually transmitted infections (STI) and their contribution to adverse birth outcomes (ABO) in sub-Saharan Africa (SSA). We performed a case–control study to assess the prevalence of STI and their association with ABO among women attending Queen Elizabeth Central Hospital, Blantyre, Malawi.

**Methods:**

A composite case definition for ABO included stillborn, preterm and low birthweight infants and infants admitted to neonatal intensive care unit within 24 hours of birth. Following recruitment of an infant with an ABO, the next born healthy infant was recruited as a control. Multiplex PCR for *Neisseria gonorrhoeae* (NG), *Chlamydia trachomatis* (CT) and *Trichomonas vaginalis* (TV) was performed on maternal vaginal swabs. HIV and syphilis status was determined on maternal and infant serum. For syphilis, we used combined treponemal/non-treponemal rapid point-of-care tests in parallel with rapid plasma reagin tests, PCR for *Treponema pallidum* and clinical parameters to diagnose and stage the infection. We compared STI positivity between cases and controls.

**Results:**

We included 259 cases and 251 controls. Maternal prevalence of STI was 3.1%, 2.7% and 17.1% for NG, CT and TV, respectively. Maternal prevalence of untreated syphilis was 2.0% and 6.1% for early stage and late/unknown stage, respectively; prevalence of treated syphilis was 2.7%. The HIV prevalence was 16.5%. HIV infection significantly increased the odds for ABO (OR=3.31; 95% CI 1.10 to 9.91) as did NG positivity (OR=4.30; 95% CI 1.16 to 15.99). We observed higher rates of ABO among women with untreated maternal syphilis (early: OR=7.13; 95% CI 0.87 to 58.39, late/unknown stage: OR=1.43; 95% CI 0.65 to 3.15). Maternal TV and CT infections were not associated with ABO.

**Conclusion:**

STI prevalence among pregnant women in Malawi is comparable to other SSA countries. HIV, NG and untreated syphilis prevalence was higher among women with ABO compared with women with healthy infants.

**WHAT IS ALREADY KNOWN ON THIS TOPIC**

- Common, curable sexually transmitted infections (STI) usually present with mild to no symptoms and are commonly missed during routine antenatal care check-ups in regions that rely on syndromic management.

**WHAT THIS STUDY ADDS**

- Using sensitive laboratory-based diagnostic tools we showed that HIV, *Neisseria gonorrhoeae* (NG) and untreated syphilis prevalence was higher among women with adverse birth outcomes compared with women with healthy infants.

**HOW THIS STUDY MIGHT AFFECT RESEARCH, PRACTICE OR POLICY**

- Pregnancy represents an opportunity for STI screening with benefits for mother and infant. Our study findings warrant the need to develop affordable (near) point-of-care diagnostics for NG. High rates of syphilis and HIV coinfections underscore the need for dual screening of pregnant women and their partners using existing frameworks.

## Introduction

 Sexually transmitted infections (STI) are widespread globally and being infected during pregnancy has been associated with up to four times increased odds of preterm birth, low birth weight (LBW) or even stillbirth.^1^,^4^ Importantly, the four most common non-viral STIs are curable: syphilis (*Treponema pallidum*), gonorrhoea (*Neisseria gonorrhoeae*; NG), chlamydia (*Chlamydia trachomatis*; CT) and trichomoniasis (*Trichomonas vaginalis*; TV). In HIV-infected women, coinfections with STIs may increase the risk of mother-to-child transmission of HIV,^5^ while in HIV-uninfected individuals, STIs increase susceptibility to HIV through the activation of proinflammatory cytokines associated with HIV acquisition.^6^

Untreated maternal syphilis is one of the leading causes of preventable stillbirth, with over 200 000 annual cases globally and the highest burden in Africa.^7 8^ Syphilis treatment is highly affordable and adverse pregnancy outcomes can be prevented if treatment is initiated before the third trimester.^9 10^ To reduce the burden of congenital syphilis and mother-to-child transmission of HIV, the WHO recommends screening for HIV and syphilis during pregnancy and the provision of respectively antiretroviral therapy or benzathine penicillin to individuals who test positive. For the detection and treatment of other urogenital infections, the WHO recommends syndromic case management for low-resource settings where laboratory facilities or trained staff are unavailable. Common symptoms of STIs include vaginal discharge, urethral discharge in men, genital ulcers and abdominal pain, but patients will be asymptomatic in many cases. It is estimated that the syndromic approach fails to identify up to 80% of chlamydial and gonococcal infections and up to 70% of trichomoniasis.^11^,^13^

Southern Africa has the highest global burden of STI,^14^ but a recent systematic review highlighted that epidemiological data on the burden of STI in pregnancy are lacking.^14^ Malawi, a low-income country in Southeast Africa, has one of the highest HIV prevalence rates globally, with 7.7% of adults being HIV positive and reproductive-age women (15–49 years) being disproportionally affected.^15^ STI prevalence data among pregnant women for Malawi and their contribution to adverse birth outcomes (ABO) are very limited.^16^ This study aims to determine the laboratory-confirmed prevalence of STI during the antenatal period in Blantyre and assess the association with ABO in this setting.

## Methods

This was a prospective case—control study of STI prevalence and associated ABO. It was performed at Queen Elizabeth Central Hospital (QECH) in Blantyre, Malawi, which has an annual birth cohort of ~10 000 babies. QECH is the referral hospital for southern Malawi and as such represents a relatively high-acuity setting in which 20–30% of infants born are premature, have LBW or require admission to the neonatal intensive care unit (NICU). All women who delivered at or presented to QECH within 48 hours after birth were eligible for inclusion in the study. Eligible ‘case’ infants were identified from the ward registers and included any infant who was stillborn, preterm, LBW defined as weighing <2500 g or who had been admitted to the NICU in the first 48 hours of life. By including the NICU we were able to further capture infants who develop clinical syndromes consistent with symptomatic congenital STI infection (conjunctivitis, pneumonia, congenital syphilis, etc). As diagnosis of these syndromes among infants may not be available at time of recruitment due to limited routine laboratory diagnostics, submission to NICU within the first 24 hours after birth (regardless of reason for submission) was regarded as case. In the absence of consistent antenatal gestational age assessment, prematurity was assessed using a predefined clinical algorithm (see online supplemental file 1 for prematurity algorithm). For each case infant recruited, the study team identified the next healthy, term baby admitted to the postnatal ward who was eligible to be a control. All participants gave written informed consent.

We aimed to recruit 650 cases and 650 controls to give 80% power to detect an OR of 1.5 with alpha=0.05, assuming a baseline prevalence of 15%. A sample size of at least 364 individuals would allow estimation of prevalence for each STI with a ±5% margin of error.

Study staff collected vaginal swabs and blood samples from all women at the time of recruitment. Samples from infants, including blood samples, nasopharyngeal (NP), skin or eye swabs, were only collected if clinically indicated, that is, in syphilis-exposed infants or those with neonatal eye infections. Clinical data, including antenatal screening and obstetric complications, were collected from participant’s health passports. Additional demographic and sexual health data were collected through face-to-face data interview on electronic case report forms. Social economic status (SES) was self-reported using the ‘Step question: on a scale of 1 to 6 (1 the poorest and 6 the richest) where do you see yourself?’ and by calculating the probability of a household living below the poverty line (<$2/day), which is based on the ‘Step’ questions of poverty indicators, including number of household members, educational level of head of household, sleeping arrangements of head of household, access to electricity, having a bank account, adequacy of food and/or clothing, ownership of a HiFi, sofa and/or iron. If HIV status was unknown, women were referred to the Lighthouse Trust at QECH for same-day HIV testing. In addition, HIV-reactive women, defined as those who were newly diagnosed during the study or those with a prior documented diagnosis, had viral load and CD4 testing performed. All women were screened for syphilis at the point of care using the DPP Screen and Confirm Assay and in parallel in the laboratory using the rapid plasma reagin (RPR) test. Women diagnosed with HIV or syphilis infection during the study were referred for treatment and follow-up in line with local guidelines.

Qualitative and quantitative syphilis diagnoses were performed using the DPP Syphilis Screen & Confirm Test Kit (Chembio, New York, USA) and the Arkray Rapid Plasma Reagin Test Kit (Arkray, Kyoto, Japan) according to the manufacturer’s protocol. The DPP assay qualitatively detected antibodies to the non-treponemal and treponemal antigens in 5 μL of serum. The RPR is a quantitative non-treponemal test. To determine antibody titre, 50 μL of serum was serially diluted (1:2, 1:4, 1:8, 1:16). Women with positive treponemal/non-treponemal serology were further classified into three categories: (1) early untreated maternal syphilis (primary, secondary and early latent syphilis of not more than 2 years of duration); (2) untreated maternal syphilis, late or unknown stage; and (3) treated maternal syphilis, stage unknown (three injections of benzathine penicillin with the last dose given at least 30 days prior to delivery), using a combination of serology, PCR results (see below) and information on (history of) clinical symptoms, screening and/or treatment (see online supplemental file 2). We considered infants to have confirmed congenital syphilis if their secretions tested PCR positive for *T. pallidum* and/or their RPR titres were fourfold higher than the maternal RPR titre.

Maternal vaginal and infant NP or eye/skin swabs were collected and stored in 1.5 mL universal transport media at −80°C prior to processing. DNA from swab samples was extracted using the QIAamp UCP Pathogen Kit (Qiagen, Hilden, Germany) according to the manufacturer’s protocol. DNA yield was determined by the Qubit 2.0 Fluorometer and the Qubit dsDNA HS Assay Kit (Thermo Fisher, Waltham, USA) according to the manufacturer’s protocol. The maternal vaginal and infant NP and eye/skin swab samples were tested using real-time STI multiplex PCR for NG, TV and CT.^17^ Singleplex PCR for *T. pallidum*^18^ was performed on infant swabs (NP or eye/skin) collected from infants born to syphilis-seropositive mothers. In addition, *T. pallidum* singleplex PCR was performed on vaginal swabs from mothers with positive syphilis serology and mothers reporting a genital ulcer during this pregnancy. All amplification reactions were performed on an Applied Biosystems 7500 Fast Real-Time PCR System or QuantStudio 7 Flex Real-Time PCR System. Positive and negative controls were included with every run. Samples with Ct≤40 were considered positive.

We compared demographic and clinical characteristics between cases and controls using Mann-Whitney U tests for continuous variables, the χ^2^ test for categorical variables and the Fisher’s exact test when the expected cell count was less than 5. Similarly, we compared demographic and clinical characteristics between STI-positive and STI-negative women. To assess whether a diagnosis of STI at delivery was independently associated with an ABO, we performed a multivariable logistic regression analysis. Covariates included in the final model were based on a directed acyclic graph (DAG) designed to select for confounding variables (see online supplemental file 3). The variables included in the DAG were chosen a priori and were based on the literature, pilot data and clinical observation. Linear variables were categorised according to the median value, and where collinearity was observed, the variable with the lowest p value was chosen. Data were analysed using SPSS V.27. Prevalence estimates were generated using the R ‘prevalence’ package V.4.2.2.

## Results

Between August 2021 and March 2022, we enrolled 510 out of 951 eligible women, of whom 259 were enrolled as cases and 251 as controls. Of the case infants, 77 (29.7%) were premature, 71 (27.4%) were LBW, 179 (69.1%) were admitted to the NICU and 38 (14.7%) were stillborn, with many case infants having more than one of these conditions (online supplemental file 4). The cases and controls were similar with respect to the main demographic and obstetric variables (table 1). In terms of infectious complication in pregnancy, 21 (4.1%) participants reported having had malaria during their pregnancy, cases n=8 (3.1%) versus controls n=13 (5.2%). 310 (60.8%) women were screened for syphilis during their pregnancy according to their health passports, of which 27 (27/310; 8.7%) reported a positive result and 23 (23/27; 85.2%) of these women received treatment for syphilis during the pregnancy, of whom 10 (14/27; 51.8%) received ‘full’ treatment, that is, three benzathine penicillin injections of which the last dose was given at least 30 days prior to delivery. Our study detected an additional 28 maternal syphilis cases of which six had previously been screened negative and 10 were categorised as early untreated syphilis infection. Most women (491/510; 96.3%) had their HIV status reported in their health passports, of whom 84 (16.5%) had a known HIV infection. With respect to STI-related symptoms reported during this pregnancy, vaginal discharge was the most reported (41/510; 8.0%), followed by dysuria (29/510; 5.7%), genital ulcers (21/510; 4.1%) and abdominal pain (17/510; 3.3%). Cases were more likely to report dysuria (7.7% vs 3.6%; p=0.044) and abdominal pain (4.6% vs 2.0%; p=0.097) compared with controls. See table 1.

**Table 1:** Demographic and clinical characteristics of maternal study participants stratified by neonatal outcome

	Totaln=510 (%)	Casesn=259 (%)	Controlsn=251 (%)	P value	Unadjusted OR(95% CI)	Adjusted* OR(95% CI)
**Demographics**						
Age (median, IQR)	24.0(19.5–30.0)	23.0(19.0–29.5)	25.0(20.0–32.0)	0.103		
<25 years	264 (51.8)	144 (55.6)	120 (47.8)	0.051	1.37 (0.97 to 1.94)	0.98 (0.60 to 1.59)
≥25 years	246 (48.2)	115 (44.4)	131 (52.2)		Ref	Ref
Parity (median, IQR)	1 (1–3)	1 (1–3)	2 (1–3)	0.183		
Gravidity†				**0.019**	1.55 (1.07 to 2.25)	1.89 (1.13 to 3.17)
Paucigravidae	335 (65.8)	183 (70.7)	152 (60.8)			
Multigravidae	174 (34.2)	76 (29.3)	98 (39.2)			
Marital status				0.657		
Married/cohabiting	454 (89.9)	233 (90.0)	221 (88.0)			
Unmarried	56 (10.1)	26 (10.0)	30 (12.0)			
Educational level				0.708		
Primary or less	308 (60.4)	165 (63.7)	143 (57.0)			
Secondary	162 (31.8)	76 (29.3)	86 (34.3)			
Tertiary	40 (7.8)	18 (6.9)	22 (8.8)			
Occupation				0.289		
Home maker	322 (63.1)	169 (65.3)	153 (61.0)			
Employed	113 (22.2)	50 (19.5)	63 (24.8)			
Student	25 (4.9)	14 (5.4)	11 (4.4)			
Unemployed	43 (8.4)	25 (9.8)	18 (7.2)			
Household SES‡						
Self-recorded ‘Step’ (median, IQR)	2 (2–3)	2 (2–3)	2 (2–3)	0.109	1.18 (0.98 to 1.43)	0.84 (0.69 to 1.04)
Probability of living below Malawi poverty line	0.03(0.0–0.17)	0.03(0.0–0.13)	0.03(0.0–0.21)	0.713		
**Obstetrics**						
Mode of delivery				0.808		
Unassisted vaginal birth	439 (86.1)	222 (85.7)	217 (86.5)			
Caesarean section (elective and emergency)	71 (13.9)	37 (14.3)	34 (13.5)			
More than 4 ANC visits	160 (31.4)	88 (34.9)	72 (29.4)	0.408		
Gestational diabetes§	1	0	1	0.355		
Gestational hypertension§	44 (8.6)	19 (7.4)	25 (9.8)	0.337	0.71 (0.38 to 1.34)	0.87 (0.45 to 1.67)
Malaria§	21 (4.1)	8 (3.1)	13 (5.2)	0.235		
Received sulfadoxine-pyrimethamine preventative treatment	444 (87.1)	226 (87.3)	218 (86.9)	0.891		
**Sexuallytransmittedinfections**						
HIV status				**0.044**		
Reactive	84 (16.5)	48 (18.5)	36 (14.3)		2.99 (1.06 to 8.44)	3.31 (1.10 to 9.91)
Unreactive	407 (79.8)	197 (76.1)	210 (83.7)		Ref	Ref
Unknown	19 (3.1)	14 (5.4)	5 (2.0)			
On ART (% of HIV positives)	83 (98.8)	47 (97.9)	36 (100)			
Syphilis status				**0.047**		
Early untreated maternal syphilis	10 (2.0)	9 (3.5)	1 (0.4)		9.28 (1.12 to 73.86)	7.13 (0.87 to 58.39)
Untreated maternal syphilis (late/unknown stage)	31 (6.1)	19 (7.3)	12 (4.8)		1.66 (0.76 to 3.44)	1.43 (0.65 to 3.15)
Treated maternal syphilis	14 (2.7)	7 (2.7)	7 (2.8)		1.03 (0.36 to 2.99)	1.05 (0.34 to 3.25)
Seronegative or does not meet criteria for syphilis diagnosis	445 (89.2)	224 (86.5)			Ref	Ref
*Neisseriagonorrhoeae* positive PCR¶	16 (3.1)	13 (5.0)	3 (1.2)	**0.039**	4.39 (1.24 to 15.59)	4.30 (1.16 to 15.99)
*Chlamydia trachomatis* positive PCR¶	14 (2.7)	4 (1.5)	10 (4.0)	0.093	0.38 (0.12 to 1.23)	0.28 (0.08 to 0.99)
*Trichomonas vaginalis* positive PCR¶	87 (17.1)	41 (15.8)	46 (18.3)	0.658	0.84 (0.53 to 1.34)	0.81 (0.49 to 1.34)
STI-related symptoms during this pregnancy						
Vaginal discharge	41 (8.0)	24 (9.3)	17 (6.8)	0.300		
Dysuria	29 (5.7)	20 (7.7)	9 (3.6)	**0.044**		
Abdominal pain	17 (3.3)	12 (4.6)	5 (2.0)	0.097		
Genital ulcers	21 (4.1)	12 (4.6)	9 (3.6)	0.552		
Symptoms treated during this pregnancy	26 (5.1)	15 (5.8)	11 (4.4)	0.226		

Bold numbers represent significant (p<0.05) associations comparing STI -positive participants to STI -negative participants, using Mann-Whitney U tests for continuous variables, theChi-squareχ2 test for categorical variables and thefFisher’s exact test when the expected cell count was less than 5.

* The multivariable model accounted for maternal age, gravidity (referenced to ≤2 previous pregnancies), gestational hypertension, SES (increase in step) and maternal HIV status.

† Odds ratiosORs were calculated using paucigravidae (1st and 2nd pregnancyies) as reference.

‡ SES: social economic status (SES), self-reported using the ‘Step question: on a scale of 1 to 6 (1 the poorest and 6 the richest) where do you see yourself?’ and by calculating the probability of a household living below the poverty line (<$2$/day), which is based on a combination of poverty indicators, including number of household members, educational level of head of household, sleeping arrangements of head of household, access to electricity, having a bank account, adequacy of food and/or clothing, ownership of a HiFi, sofa and/or iron.

§ As reported in health passports.

¶ 507/510 (99.4%) of the recruited mothers provided a vaginal swab for multiplex PCR testing; missing STI results (n=2 cases, n=1 control) were regarded as negatives.

ANCantenatal careARTantiretroviral therapySTIsexually transmitted infection

In total, we collected 507 maternal vaginal swabs (99.4% of participants), 146 infant NP swabs, 3 infant skin swabs and 1 infant eye swab. The three participants with missing vaginal swabs (n=2 cases, n=1 control) were included in all further analyses and their CT/NG/TV results were treated as negative. The overall prevalence estimate of NG, CT and TV on maternal swabs collected at recruitment was 3.1% (95% CI 1.6% to 4.6%), 2.7% (95% CI 1.3% to 4.1%) and 17.1% (95% CI 13.8% to 20.3%), respectively. NG positivity was significantly higher among cases compared with controls (13/259; 5% vs 3/251; 1.2%, p=0.039), whereas there was no statistically significant difference for TV (41/259; 15.8% vs 46/251; 18.3%, p=0.658) and CT (4/259; 1.5% vs 10/251; 4.0%, p=0.093), both of which were slightly more prevalent among controls than among cases. HIV prevalence was higher among cases than controls (48/259; 18.5% vs 36/251; 14.3%, p=0.044), as was untreated syphilis (early: 9/259; 3.5% vs 1/251; 0.4%, p=0.012, late/unknown: 19/259; 7.3% vs 12/251; 4.8%, p=0.227). Treated syphilis prevalence was similar among cases and controls, respectively, 7/259; 2.7% vs 7/251; 2.8%, p=0.953. 117 infants in our study had NP or eye swabs performed due to a clinical indication (ie, if they had neonatal pneumonia or eye infection), and 5 (4.2%) of these infants tested positive for *T. pallidum* (4 NP and 1 eye swab)*,* 3 (2.6%) tested positive for NG and 8 (6.8%) tested for CT in the nasopharynx. See onlinesupplemental tables 1  2.

**Figure 1:**
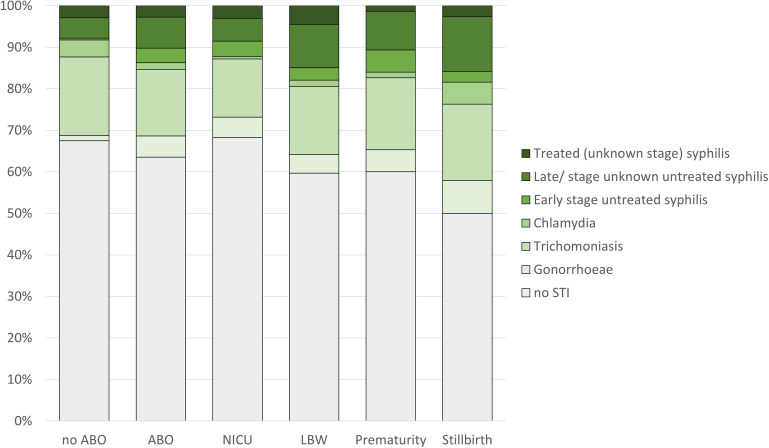
Proportion of adverse birth outcomes (ABO) testing positive for sexually transmitted infection (STI). ABO included the composite outcome of low birth weight (LBW), prematurity or admission to neonatal intensive care unit (NICU), and controls (no ABO) were defined as next born healthy infant. Mother/infant dyads were recruited at Queen Elizabeth Central Hospital, Blantyre, Malawi, between August 2021 and March 2022.

In univariable analysis, a diagnosis of NG was associated with the composite ABO outcome (OR=4.39; 95% CI 1.24 to 15.59), as was a diagnosis of early untreated syphilis (OR=9.28; 95% CI 1.12 to 73.86) and HIV positivity (OR=2.99; 95% CI 1.06 to 8.44), but no statistically significant associations were observed for the individual ABO outcomes. No associations were observed for TV or CT with LBW, prematurity, stillbirth, NICU admission alone or the composite ABO outcome. Untreated maternal syphilis (late/unknown stage) increased the odds for ABO, but not statistically significantly so (OR=1.66; 95% CI 0.76 to 3.44). Treated maternal syphilis was not associated with ABO. In a multivariable analysis where maternal age, gravidity (referenced to ≤2 previous pregnancies), gestational hypertension, SES (increase in step) and maternal HIV status were accounted for, HIV infection was significantly associated with having an ABO (OR=3.31; 95% CI 1.10 to 9.91) as was NG positivity (OR=4.30; 95% CI 1.16 to 15.99). Untreated maternal syphilis (early: OR=7.13; 95% CI 0.87 to 58.39, late/unknown stage: OR=1.43; 95% CI 0.65 to 3.15) increased the odds for ABO but the association was not statistically significant. Maternal TV and CT infections were not associated with ABO. See tables1  2 and figure 1.

**Table 2:** Neonatal outcome, maternal HIV status and STI-related symptoms associated with maternal STI status

	Total n=510 (%)	Any non-routinely screened STI (NG/TV/CT)n=106 (%)	NGn=16 (%)	TVn=87 (%)	CTn=14 (%)	Early-stage syphilisn=10 (%)	Late/unknown-stage untreated syphilisn=31 (%)	Treated syphilisn=14 (%)
Adverse birth outcome	259 (50.8)	51 (48.1)	**13(82.2)**	41 (47.1)	4 (28.6)	**9(90.0)**	19 (61.3)	7 (50.0)
Prematurity (<37 weeks’ gestation)	77 (15.1)	16 (15.1)	4 (25.0)	13 (14.9)	1 (7.1)	4 (40.0)	7 (22.6)	1 (7.1)
Low birth weight (<2500 g)	71 (13.9)	13 (21.1)	3 (18.8)	11 (12.6)	1 (7.1)	2 (20.0)	7 (22.6)	3 (21.4)
Admission to NICU	179 (35.1)	32 (30.2)	8 (50.0)	25 (28.7)	2 (14.3)	6 (60.0)	9 (64.3)	5 (35.7)
Stillbirth	38 (7.5)	9 (8.5)	3 (18.8)	7 (8.0)	2 (14.3)	1 (10.0)	5 (16.1)	1 (7.1)
Congenital syphilis	5 (1.0)					1 (5.9)	4 (1.7)	0 (0.0)
HIV reactive	84 (16.5)	21 (19.8)	4 (25.0)	17 (19.5)	1 (7.1)	**5(50.0)**	**10(32.3)**	**9(64.3)**
STI-related symptoms								
Vaginal discharge	41 (8.0)	12 (11.3)	3 (18.8)	10 (11.5)	1 (7.1)	2 (20.0)	7 (22.6)	2 (14.3)
Dysuria	29 (5.7)	6 (5.7)	**3(18.8)**	3 (3.4)	0 (0.0)	0 (0.0)	7 (22.6)	0 (0.0)
Abdominal pain	17 (3.3)	2 (1.9)	**2(12.5)**	0 (0.0)	0 (0.0)	2 (20.0)	4 (12.9)	0 (0.0)
Genital ulcers	21 (4.1)	6 (5.7)	2 (12.5)	4 (4.6)	0 (0.0)	4 (40.0)	1 (3.2)	0 (0.0)

Bold numbers represent significant (p<0.05) associations comparing STI -positive participants to STI -negative participants, using Chi-squareχ2 tests for comparing categorical variables and thefFisher’s exact test when the expected cell count was less than 5.

CT*Chlamydia trachomatis*NG*Neisseria gonorrhoeae*NICUneonatal intensive care unitSTIsexually transmitted infectionTV*Trichomonas vaginalis*

**Figure 2:**
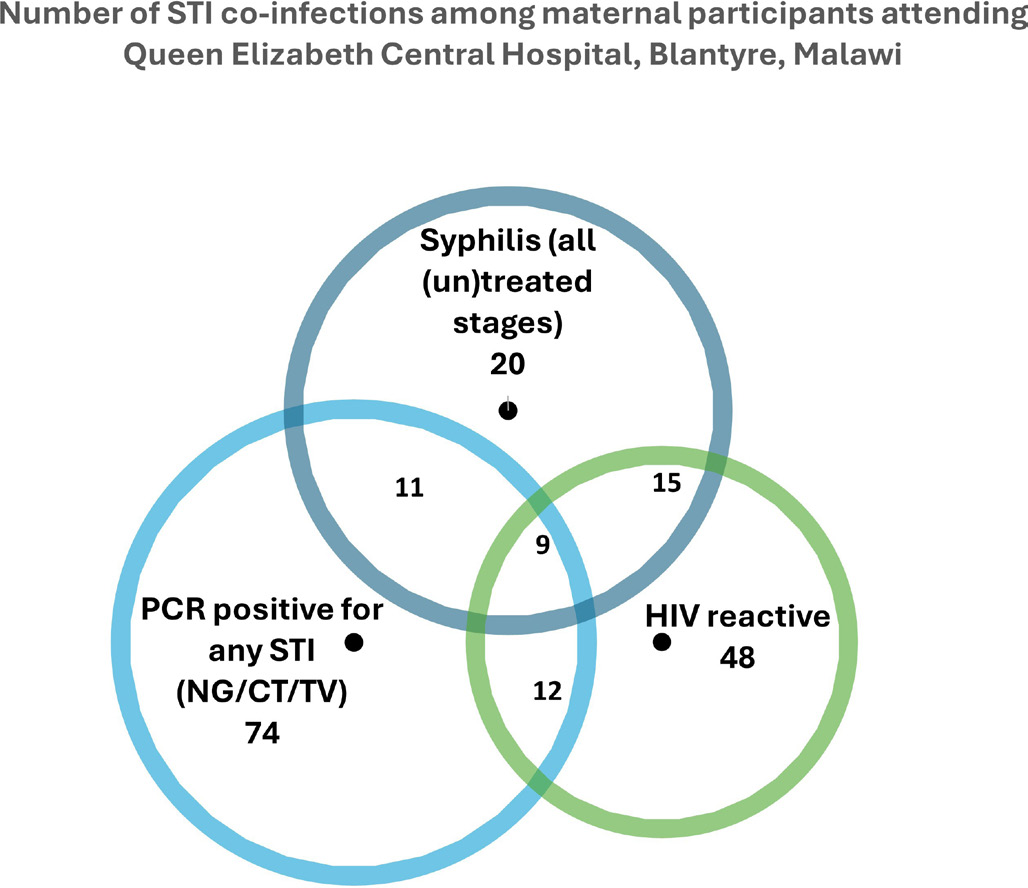
Number of sexually transmitted infection (STI) coinfections among maternal participants. Mother/infant dyads were recruited at Queen Elizabeth Central Hospital, Blantyre, Malawi, between August 2021 and March 2022. CT, *Chlamydia trachomatis*; NG, *Neisseria gonorrhoeae*; TV, *Trichomonas vaginalis.*

Regarding factors associated with common curable STI positivity, HIV seropositivity was associated with having syphilis regardless of stage or treatment status (5/10; 50.0%, 10/31; 32.3% and 9/14; 64.3% of women with respectively early, late/unknown stage and treated syphilis were HIV positive; p<0.001), whereas NG, TV and CT infections did not associate with HIV status. Dysuria was positively associated with NG infections (3/16; 18.8% of women testing positive for NG reported dysuria compared with 26/491; 5.3% of women testing negative for NG; p=0.023) as was abdominal pain (2/16; 12.5% vs 15/491; 3.1%, p=0.039). Reporting genital ulcers and vaginal discharge during this pregnancy was not associated with any of the infections. See table 2 and figure 2.

## Discussion

The prevalence data reported in this study are line with the pooled estimates for the Southern African region^14^ and similar to the STI prevalence reported by Chaponda *et al*^19^ among pregnant women attending antenatal care (ANC) in Zambia neighbouring country. Like Chaponda *et al,* we observed that very few women testing positive for STI reported STI-like symptoms. We report similarly high syphilis rates to the Zambian cohort and despite syphilis screening being mandatory in Malawi, only 60.8% of women had records of being screened for syphilis during this pregnancy and a minority of women (n=14; 2.7%) had records of completing treatment in line with the national guidelines in this pregnancy. In contrast, only 19 women (3.1%) were unaware of their HIV status and we detected just one new HIV case. All women with known HIV infection were on antiretroviral therapy. These results highlight the inadequacy of syndromic diagnostic methods, the importance of repeat syphilis evaluation at delivery and the urgent need for improved integration of syphilis testing in pregnancy with existing HIV testing strategies.

In keeping with the existing literature, ABOs were more common among women with HIV, NG and untreated syphilis in Malawi,^4 20^ and support interventions which would reduce the burden of these diseases in pregnancy. We observed high rates of HIV/syphilis coinfections, and improved uptake of dual testing for syphilis and HIV as recommended in the WHO strategy for triple elimination of HIV, syphilis and hepatitis B is urgently needed.^21^ Benzathine penicillin stockouts are common in Malawi and may explain the low treatment coverage observed in our study. Conversely, all HIV-positive pregnant women were receiving highly active antiretroviral therapy (HAART). Integration of funding and procurement of benzathine penicillin with HAART supplies is warranted. A recent meta-analysis on the association between NG and ABO concluded that women with NG infections were more likely to experience ABO and that the association with preterm birth was stronger for low-income to middle-income countries compared with high-income countries. The underlying mechanism is not fully understood but NG may cause low-grade inflammation affecting both the placenta and fetal membranes leading to chorioamnionitis and preterm birth.^22^ Our data suggest that NG is linked to ABO in Malawi and that point-of-care diagnostics for this pathogen could reduce ABO. In support of this, a large cluster randomised cross-over trial in Papua New Guinea demonstrated the effect of timely antenatal diagnosis and treatment of NG infections in reducing ABO.^23^ Additionally, interventions aimed at reducing risk of malaria infection during pregnancy may beneficially affect the burden of STI in relation to ABO.^24 25^ Intermittent preventative treatment using sulfadoxine-pyrimethamine (IPTp-SP) has been shown to exhibit a dose–response protective effect against ABO, and as this effect is strongest among women with malaria and/or STI, it has been suggested to have a broad-range effect decreasing malaria parasite load and partially protect against numerous gram-positive and gram-negative bacteria.^24^ Our study did not detect an association with ABO for TV or CT. This is in contrast to evidence from high-income, low-TV prevalence countries which has reported a positive association between TV infection and ABO^26^ but in keeping with limited prior evidence from the sub-Saharan Africa (SSA) region (low income to middle income, high TV prevalence) which fails to support an association in this setting.^27 28^ Clinical trial evidence from Uganda and Malawi for the effect of TV treatment during pregnancy showed conflicting results and overall did not support this intervention.^29^,^32^ A recent review concluded that there was weak to moderate evidence to suggest that CT infections are associated with adverse perinatal outcomes,^33^ and in our study we even found a negative association with ABO. Perhaps the strongest impact of CT infections on reproductive health may be at the fertility level, particularly the risk for tubal infertility seems to increase with (previous) CT infections.^3^ Despite the lack of association with ABO, pregnancy is a valuable point of contact between young women and the health service in low-income settings with a high burden of STI and presents an opportunity for a holistic approach to screening and treatment of these infections.

This is the largest published study to date using multiplex PCR for STI among pregnant women in SSA. We collected adequate clinical detail to classify maternal syphilis cases, assess syndromic diagnosis and accurately classify ABOs. The major limitation of the study was poor recruitment. Almost half of the eligible women declined to take part in the study. We did not formally document reasons for refusal, but anecdotally we noted that many women refused to take part on account of not having permission from their husbands and beliefs about syphilis being treatable by traditional rather than clinical medicine. To counter these rumours, we implemented ward sensitisation sessions two times a week during the recruitment period. In line with other studies in Malawi, pregnant women were anxious about blood samples being taken from them or their babies. The resulting suboptimal recruitment compromises our ability to make definitive statements on many of the associations. Additionally, we lacked information on the number of IPTp-SP doses administered during pregnancy which may have led to an underestimation of the attributable fraction of STI to ABO in our setting. Moreover, we sampled from a single referral hospital in a relatively high-acuity setting where 20–30% of infants born have an adverse outcome and the attributable fraction of STIs on ABO may be difficult to tease out. Moving forward, STI prevalence data need to be collected from multiple ANC and STI clinics to have better estimate of STI rates in both urban and rural settings.

## Conclusion

The prevalence of common, curable STI is substantial among pregnant women residing in the southern region of Malawi and is significantly underestimated using current syndromic diagnostic approaches. While enhanced near point-of-care testing for NG would lead to the diagnosis of a modifiable cause of ABOs, we would advocate for multiplex diagnostics. Pregnancy is a crucial opportunity for reproductive health education and access to STI diagnosis and treatment for young women and their partners. There have been substantial gains in the availability of prevention of mother to child transmission(PMTCT) of HIV in Malawi, and there is an urgent need for improved integration of syphilis diagnostics and treatment for pregnant women and their partners within the same framework. The widespread availability of treponemal-specific rapid point of care tests (POCT) for syphilis screening in Malawi has increased the coverage of this intervention in ANC, but this approach has limitations with respect to disease staging, assessment of cure, rational use of benzathine penicillin stocks and surveillance of congenital syphilis. Additional research on combined treponemal/non-treponemal rapid POCT is warranted to address these challenges while maintaining access.

## supplementary material

10.1136/sextrans-2024-056130 online supplemental file 1

10.1136/sextrans-2024-056130 online supplemental file 2

10.1136/sextrans-2024-056130 online supplemental file 3

10.1136/sextrans-2024-056130 online supplemental file 4

10.1136/sextrans-2024-056130 online supplemental table 1

10.1136/sextrans-2024-056130 online supplemental table 2

## Data Availability

Data are available upon reasonable request.

## References

[1] Gomez GB, Kamb ML, Newman LM (2013). Untreated maternal syphilis and adverse outcomes of pregnancy: a systematic review and meta-analysis. Bull World Health Organ.

[2] Heumann CL, Quilter LAS, Eastment MC (2017). Adverse birth outcomes and maternal Neisseria gonorrhoeae infection: a population-based cohort study in Washington state. Sex Transm Dis.

[3] Tang W, Mao J, Li KT (2020). Pregnancy and fertility-related adverse outcomes associated with Chlamydia trachomatis infection: a global systematic review and meta-analysis. Sex Transm Infect.

[4] Tsai S, Sun MY, Kuller JA (2019). Syphilis in pregnancy. Obstet Gynecol Surv.

[5] C. King C, R. Ellington S, P. Kourtis A (2013). The role of co-infections in mother-to-child transmission of HIV. Curr HIV Res.

[6] Masson L, Passmore J-AS, Liebenberg LJ (2015). Genital inflammation and the risk of HIV acquisition in women. Clin Infect Dis.

[7] Schmid G (2004). Economic and programmatic aspects of congenital syphilis prevention. Bull World Health Organ.

[8] Watson-Jones D, Changalucha J, Gumodoka B (2002). Syphilis in pregnancy in Tanzania. I. Impact of maternal syphilis on outcome of pregnancy. J Infect Dis.

[9] Terris-Prestholt F, Watson-Jones D, Mugeye K (2003). Is antenatal syphilis screening still cost effective in sub-Saharan Africa. Sex Transm Infect.

[10] Watson-Jones D, Gumodoka B, Weiss H (2002). Syphilis in pregnancy in Tanzania. II. The effectiveness of antenatal syphilis screening and single-dose benzathine penicillin treatment for the prevention of adverse pregnancy outcomes. J Infect Dis.

[11] Msuya SE, Uriyo J, Stray-Pedersen B (2009). The effectiveness of a syndromic approach in managing vaginal infections among pregnant women in northern Tanzania. East Afr J Public Health.

[12] Moodley D, Moodley P, Sebitloane M (2015). High prevalence and incidence of asymptomatic sexually transmitted infections during pregnancy and postdelivery in Kwazulu natal, South Africa. Sex Transm Dis.

[13] Mudau M, Peters RP, De Vos L (2018). High prevalence of asymptomatic sexually transmitted infections among human immunodeficiency virus-infected pregnant women in a low-income South African community. Int J STD AIDS.

[14] Nyemba DC, Haddison EC, Wang C (2022). Prevalence of curable STIs and bacterial vaginosis during pregnancy in sub-Saharan Africa: a systematic review and meta-analysis. Sex Transm Infect.

[15] UNAIDS Focus on Malawi. https://www.unaids.org/en/20190402_country_focus_Malawi.

[16] Mtove G, Chico RM, Madanitsa M (2023). Fetal growth and birth weight are independently reduced by malaria infection and curable sexually transmitted and reproductive tract infections in Kenya, Tanzania, and Malawi: a pregnancy cohort study. Int J Infect Dis.

[17] Rumyantseva T, Golparian D, Nilsson CS (2015). Evaluation of the new AmpliSens multiplex real-time PCR assay for simultaneous detection of Neisseria gonorrhoeae, Chlamydia trachomatis, Mycoplasma genitalium, and trichomonas vaginalis. APMIS.

[18] Heymans R, van der Helm JJ, de Vries HJC (2010). Clinical value of Treponema pallidum real-time PCR for diagnosis of syphilis. J Clin Microbiol.

[19] Chaponda EB, Bruce J, Michelo C (2021). Assessment of syndromic management of curable sexually transmitted and reproductive tract infections among pregnant women: an observational cross-sectional study. BMC Pregnancy Childbirth.

[20] Shinar S, Agrawal S, Ryu M (2022). Perinatal outcomes in women living with HIV-1 and receiving antiretroviral therapy-a systematic review and meta-analysis. Acta Obstet Gynecol Scand.

[21] Cohn J, Owiredu MN, Taylor MM (2021). Eliminating mother-to-child transmission of human immunodeficiency virus, syphilis and hepatitis B in sub-Saharan Africa. Bull World Health Organ.

[22] Vallely LM, Egli-Gany D, Wand H (2021). Adverse pregnancy and neonatal outcomes associated with Neisseria gonorrhoeae: systematic review and meta-analysis. Sex Transm Infect.

[23] Riddell MA, Vallely LM, Mengi A (2024). Point-of-care testing and treatment of sexually transmitted and genital infections to improve birth outcomes in high-burden, low-resource settings (WANTAIM): a pragmatic cluster randomised crossover trial in Papua New Guinea. Lancet Glob Health.

[24] Chico RM, Chaponda EB, Ariti C (2017). Sulfadoxine-pyrimethamine exhibits dose-response protection against adverse birth outcomes related to malaria and sexually transmitted and reproductive tract infections. Clin Infect Dis.

[25] Madanitsa M, Barsosio HC, Minja DTR (2023). Effect of monthly intermittent preventive treatment with dihydroartemisinin-piperaquine with and without azithromycin versus monthly sulfadoxine-pyrimethamine on adverse pregnancy outcomes in Africa: a double-blind randomised, partly placebo-controlled trial. Lancet.

[26] Silver BJ, Guy RJ, Kaldor JM (2014). Trichomonas vaginalis as a cause of perinatal morbidity: a systematic review and meta-analysis. Sex Transm Dis.

[27] Moodley D, Sartorius B, Madurai S (2017). Pregnancy outcomes in association with Stds including genital HSV-2 shedding in a South African cohort study. Sex Transm Infect.

[28] Warr AJ, Pintye J, Kinuthia J (2019). Sexually transmitted infections during pregnancy and subsequent risk of stillbirth and infant mortality in Kenya: a prospective study. Sex Transm Infect.

[29] Gray RH, Wabwire-Mangen F, Kigozi G (2001). Randomized trial of presumptive sexually transmitted disease therapy during pregnancy in Rakai, Uganda. Am J Obstet Gynecol.

[30] Kigozi GG, Brahmbhatt H, Wabwire-Mangen F (2003). Treatment of trichomonas in pregnancy and adverse outcomes of pregnancy: a subanalysis of a randomized trial in Rakai, Uganda. Am J Obstet Gynecol.

[31] Luntamo M, Kulmala T, Mbewe B (2010). Effect of repeated treatment of pregnant women with sulfadoxine-pyrimethamine and azithromycin on preterm delivery in Malawi: a randomized controlled trial. Am J Trop Med Hyg.

[32] Stringer E, Read JS, Hoffman I (2010). Treatment of trichomoniasis in pregnancy in sub-Saharan Africa does not appear to be associated with low birth weight or Preterm birth. S Afr Med J.

[33] Adachi KN, Nielsen-Saines K, Klausner JD (2021). Chlamydia trachomatis screening and treatment in pregnancy to reduce adverse pregnancy and neonatal outcomes: a review. Front Public Health.

