# Supplementary material for: Prevalence of sexually transmitted infection in pregnancy and their association with adverse birth outcomes: a case–control study at Queen Elizabeth Central Hospital, Blantyre, Malawi
Source: Sex Transm Infect. 2024 Jul 23;100(8):e056130. doi: 10.1136/sextrans-2024-056130 (PMC11671869; doi:10.1136/sextrans-2024-056130)
Supplement: online supplemental file 1 [file sextrans-100-8-s001.pdf]

## ALGORITHYM TO DETERMINE PREMATURITY( SOP-001)

For "case recruitment" This includes all women who have delivered an infant who is: stillborn (fresh or macerated), premature (< 37 weeks), low birth weight (<2500gm or 2.5kg) or have had their infant admitted to NICU in the preceding 48 hours.

A diagnosis of prematurity may be difficult to determine clinically between 34-37 weeks.

1.1.1 To determine the likelihood that an infant is preterm and their gestation the following information will be collected:

- LMP
- USS dating at any stage in pregnancy.
- Ballard score at birth.
- SFH collected during the second trimester.
- Birth weight

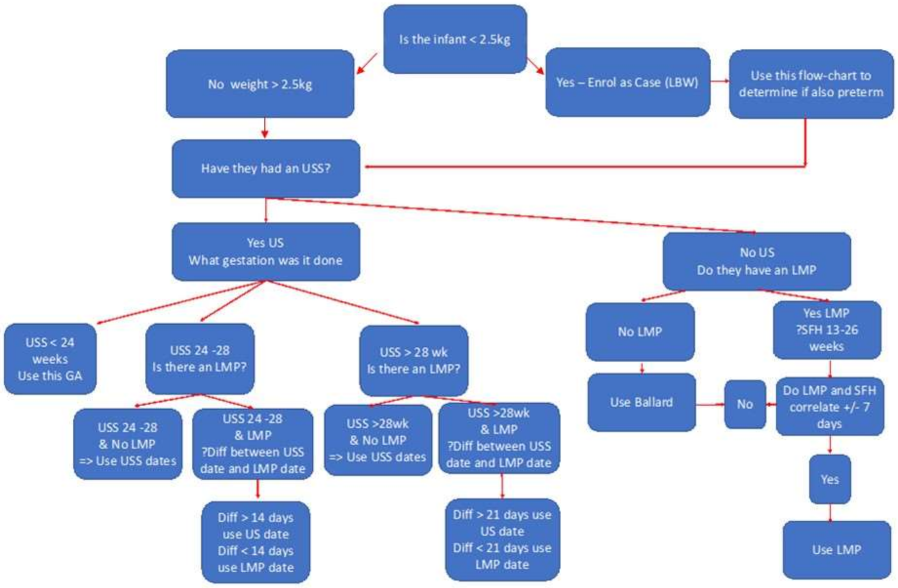


## Results

142 (28%) of included women had an US during current pregnancy (n=30 before 24 weeks), 201 (29.5%) reported LMP and 315 (62%) reported SFH. Birth weight and Ballard scores were available for all live infants.
