# Supplementary material for: Prevalence of sexually transmitted infection in pregnancy and their association with adverse birth outcomes: a case–control study at Queen Elizabeth Central Hospital, Blantyre, Malawi
Source: Sex Transm Infect. 2024 Jul 23;100(8):e056130. doi: 10.1136/sextrans-2024-056130 (PMC11671869; doi:10.1136/sextrans-2024-056130)
Supplement: online supplemental file 2 [file sextrans-100-8-s002.pdf]

Syphilis case definitions

Maternal outcome definitions:

1) **Maternal untreated early syphilis (primary, secondary and early latent syphilis of not more than two years duration)**
    a. At delivery TPPA positive and RPR positive or negative or failed AND
    b. Presence of primary chancre (genital ulcer) during this pregnancy OR
    c. Diagnosis of syphilis for first time in last two years – i.e. negative at ANC in this pregnancy +/- negative in recent pregnancy or STI check < 2 years ago

2) **Maternal untreated late (or unknown stage) syphilis (infection of more than two years duration with no clinical signs of active infection)**
    a. TPPA positive and RPR positive or negative or failed AND
    b. Had no symptoms of primary chancre AND
    c. TPPA positive at ANC1 or pre-pregnancy and no treatment OR
    d. No information regarding syphilis diagnosis in previous pregnancy or last 2 years.

3) **Maternal (unknown stage) treated syphilis**
    a. TPPA positive at ANC1 in current pregnancy AND
    b. No documentation of negative TPPA in last two years AND
    c. No documentation of symptoms consistent with syphilis in last two years AND
    d. Received 3 doses of IM BPG > 30 days prior to delivery either in this pregnancy or at the time of initial diagnosis.

4) Maternal early **treated** syphilis -> <u>none of the maternal participants met these criteria</u>
    a. TPPA positive (or Trep/NT) and RPR positive/negative or failed at delivery AND
    b. Symptoms of primary syphilis in this pregnancy AND
    c. Negative TPPA in last 2 years (including in prior pregnancy or STI clinic) AND
    d. Received 1 dose of IM BPG at least > 30 days prior to delivery in current pregnancy.

Neonatal outcomes

1. Confirmed congenital syphilis:
    a. Syphilis PCR positive from infant secretions **AND/OR**
    b. Infant TPPA positive and infant RPR > 4 fold maternal RPR

2. Probable congenital syphilis
    a. Infant TPPA positive and RPR positive but < 4 fold maternal RPR **AND/OR**
    b. Maternal early **untreated** syphilis as above without 1 dose of BPG > 30 days pre delivery **OR**
    c. Maternal late **untreated** syphilis as above without 3 doses of BPG > 30 days pre delivery
