# Supplementary figures and images for: Prevalence of sexually transmitted infection in pregnancy and their association with adverse birth outcomes: a case–control study at Queen Elizabeth Central Hospital, Blantyre, Malawi
Source: Sex Transm Infect. 2024 Jul 23;100(8):e056130. doi: 10.1136/sextrans-2024-056130 (PMC11671869; doi:10.1136/sextrans-2024-056130)

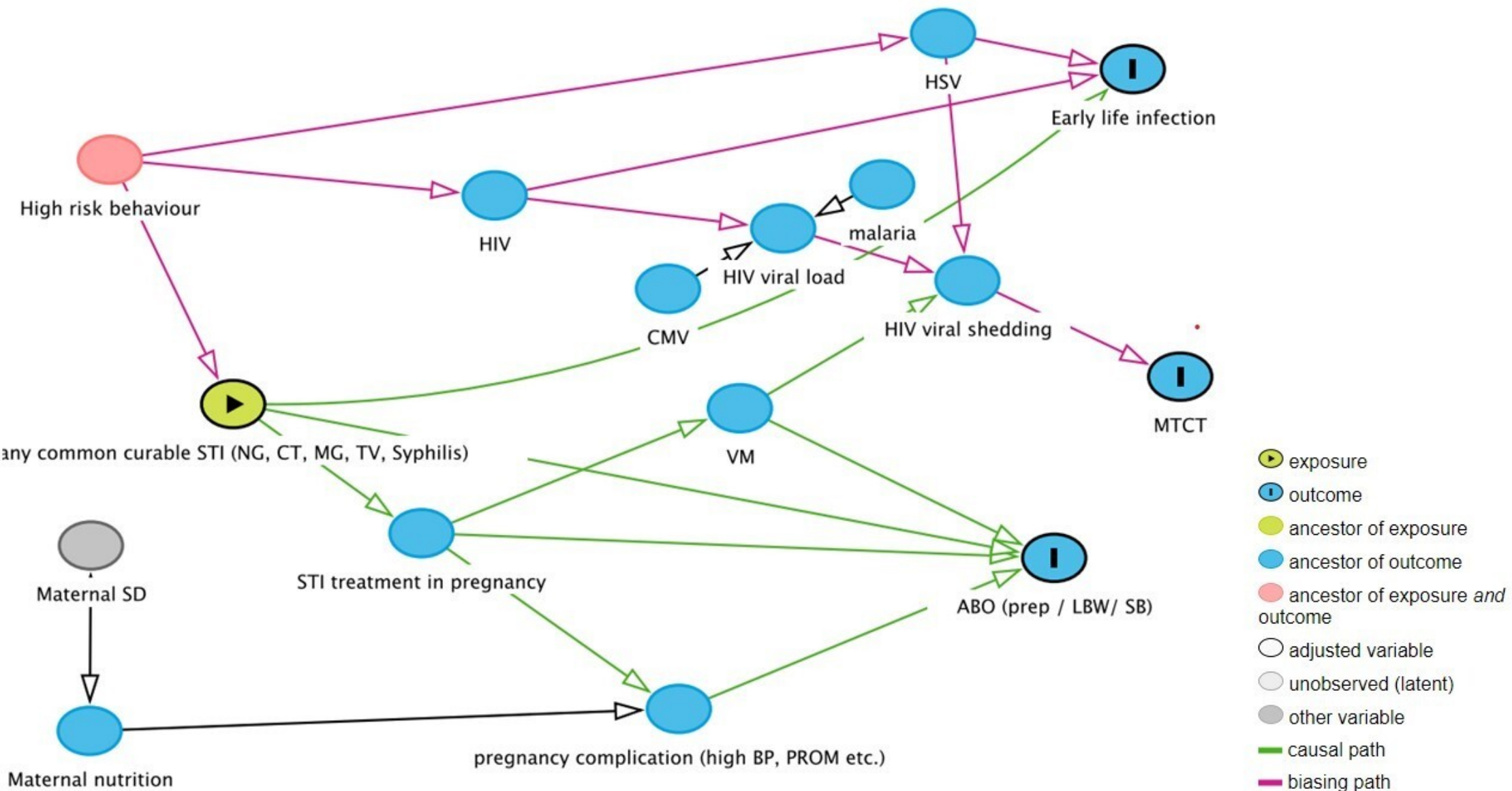

Supplement: online supplemental file 3 [file sextrans-100-8-s003.pdf]
