# Supplementary material for: Prevalence of sexually transmitted infection in pregnancy and their association with adverse birth outcomes: a case–control study at Queen Elizabeth Central Hospital, Blantyre, Malawi
Source: Sex Transm Infect. 2024 Jul 23;100(8):e056130. doi: 10.1136/sextrans-2024-056130 (PMC11671869; doi:10.1136/sextrans-2024-056130)
Supplement: online supplemental file 4 [file sextrans-100-8-s004.pdf]

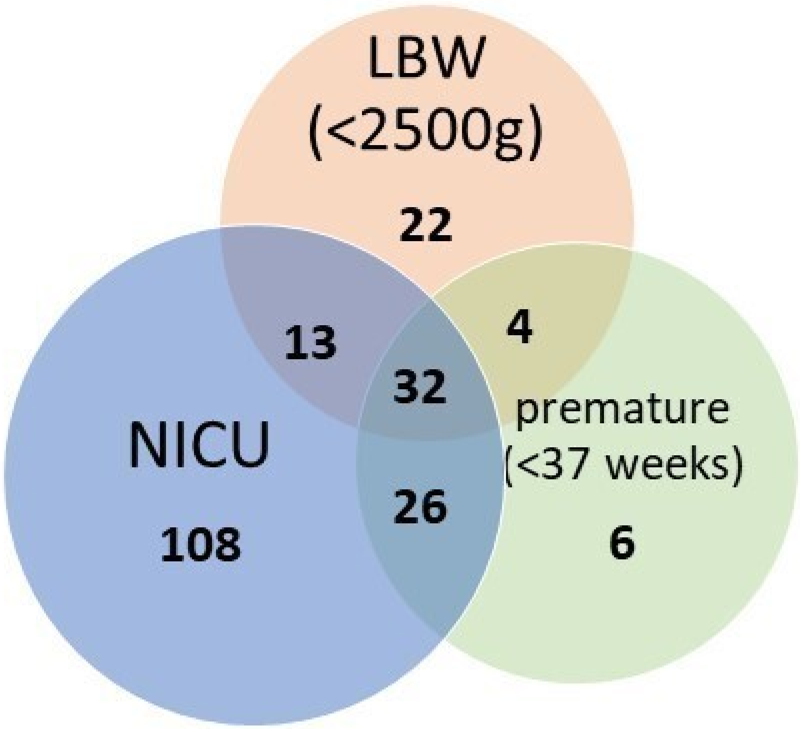

Supplementary figure. Overlap in components comprising the composite (live birth) adverse birth outcomes.
