# Supplementary material for: Prevalence of sexually transmitted infection in pregnancy and their association with adverse birth outcomes: a case–control study at Queen Elizabeth Central Hospital, Blantyre, Malawi
Source: Sex Transm Infect. 2024 Jul 23;100(8):e056130. doi: 10.1136/sextrans-2024-056130 (PMC11671869; doi:10.1136/sextrans-2024-056130)
Supplement: online supplemental table 1 [file sextrans-100-8-s005.pdf]

Supplementary Table 1. STI PCR positivity for samples collected from infants with clinical indication.

	**NP swabs n=117 (%)**	**Eye/skin swabs n=4 (%)**
**NG**	3 (2.6)	0 (0.0)
**TV**	8 (6.8)	0 (0.0)
**CT**	0 (0.0)	0 (0.0)
T. pallidum	4 (3.4)	1 (25.0)
