# Supplementary material for: Prevalence of sexually transmitted infection in pregnancy and their association with adverse birth outcomes: a case–control study at Queen Elizabeth Central Hospital, Blantyre, Malawi
Source: Sex Transm Infect. 2024 Jul 23;100(8):e056130. doi: 10.1136/sextrans-2024-056130 (PMC11671869; doi:10.1136/sextrans-2024-056130)
Supplement: online supplemental table 2 [file sextrans-100-8-s006.pdf]

Supplementary Table 2. Prevalence estimates for common curable STI among women attending Queen Elizabeth Central Hospital, Blantyre, Malawi, stratified by neonatal outcome.

	**Prevalence (95% CI)**	
	**Cases**	**Controls**
**Maternal syphilis**		
early untreated maternal syphilis	3.5% (1.2 – 5.7)	0.4% (0.0 – 1.2)
Late/stage unknown untreated maternal syphilis	7.3% (4.2 – 10.5)	4.8% (2.1 – 7.4)
Treated maternal syphilis	2.7% (0.7 – 4.7)	2.8% (0.8 – 4.8)
Neisseria gonorrhea	5.0% (2.3 – 7.7)	1.2% (0.0 – 2.5)
Chlamydia trachomatis	1.5% (0.0 – 3.0)	4.0% (1.5 – 6.4)
Trichomonas vaginalis	15.8% (11.3 – 20.3)	18.3% (13.5 – 23.1)
